# Supplementary material for: Myeloperoxidase as a Marker to Differentiate Mouse Monocyte/Macrophage Subsets
Source: Int J Mol Sci. 2022 Jul 26;23(15):8246. doi: 10.3390/ijms23158246 (PMC9330004; doi:10.3390/ijms23158246)
Supplement: Supplementary file 1 [file ijms-23-08246-s001.zip › ijms-1820153-supplementary.pdf]

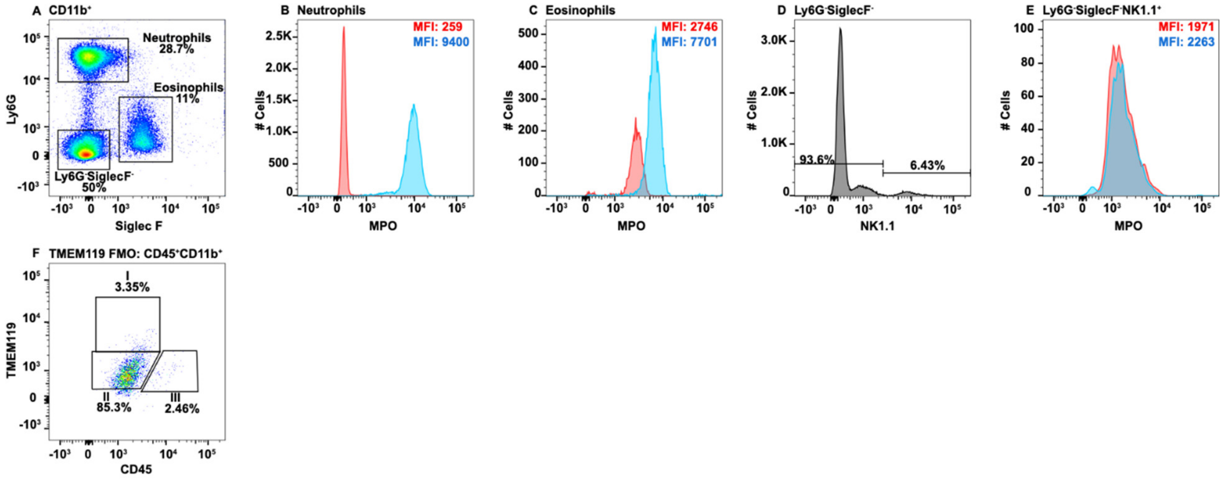


**Supplemental Figure S1**. MPO FMO controls in the spleen and TMEM119 control in the CNS. Mouse splenocytes were stained with antibodies specific to CD19, CD11b, Ly6C, Ly6G, F4/80, SiglecF and NK1.1, either with or without MPO to create MPO FMO controls (A-E). Utilizing flow cytometry, cells were gated on CD19$^{-}$CD11b$^{+}$ before eosinophils and neutrophils were separated by SiglecF and Ly6G, respectively (A). Neutrophils and eosinophils were then analyzed for MPO expression by overlaying FMO controls (red) with the same gating strategy including MPO (blue) (B-C). Ly6G$^{-}$SiglecF$^{-}$ cells were gated on NK1.1 to isolate NK cells (D). NK cells were then examined for MPO expression by overlaying with the FMO control (E). Mouse CNS cells were stained with zombie violet viability dye and antibodies specific to CD45, CD11b, Ly6C, F4/80, MPO, with or without anti-TMEM119 (F). The percent positive cells in dot plot gates and the MFI of histograms is provided. Data shown is representative of two mice.
